# Supplementary material for: Chemoorganoautotrophic lifestyle of the anaerobic enrichment culture N47 growing on naphthalene
Source: Commun Biol. 2025 Jun 4;8:856. doi: 10.1038/s42003-025-08172-y (PMC12137691; doi:10.1038/s42003-025-08172-y)
Supplement: Supplementary file 1 — Supplementary materials [file 42003_2025_8172_MOESM1_ESM.pdf]

Supplementary materials: Chemoorganoautotrophic lifestyle of the anaerobic enrichment culture N47 growing on naphthalene

Authors: Isabelle Heker[1], Christian Seitz[2], Lisa Voskuhl[1], Yachao Kong[1], Isabell Erdmann[1], Frederik Götz[1], Mohamed Hassoun[1], Claudia Huber[2], Wolfgang Eisenreich[2], Rainer U. Meckenstock[1]*

Affiliations:

[1]Faculty of Chemistry, Environmental Microbiology and Biotechnology, University of Duisburg-Essen; Essen, 45141, Germany.

[2]Bavarian NMR Center - Structural Membrane Biochemistry, Department of Bioscience, School of Natural Sciences, Technical University of Munich; Garching, 85748, Germany

*Corresponding author: Email: rainer.meckenstock@uni-due.de

Supplementary figures

Figure S1: Absorbance measurement of the aconitase and isocitrate dehydrogenase assay at 340 nm, showing the increase in NADPH.

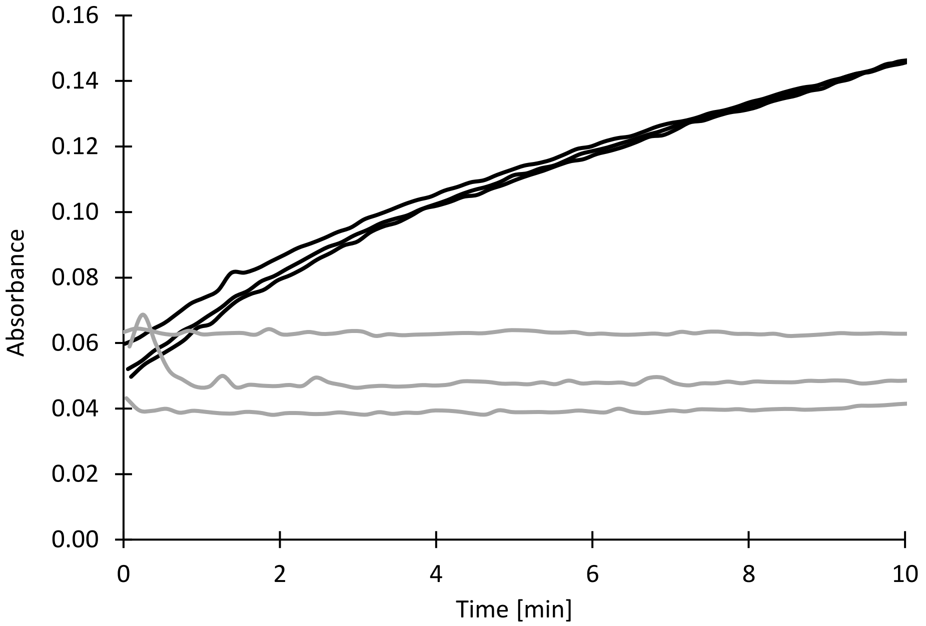

The three black lines represent the absorbance in the assays, the grey lines represent the negative controls without citrate. The assays and controls were carried out in triplicates as shown.

Figure S2: Growth of culture N47 with monitoring of CO_2 production and the absolute share of ^{13}C in the bicarbonate buffer in experiments with 10 % [U-$^{13}C_{10}$]naphthalene or 10 % $H^{13}CO_3^-$.

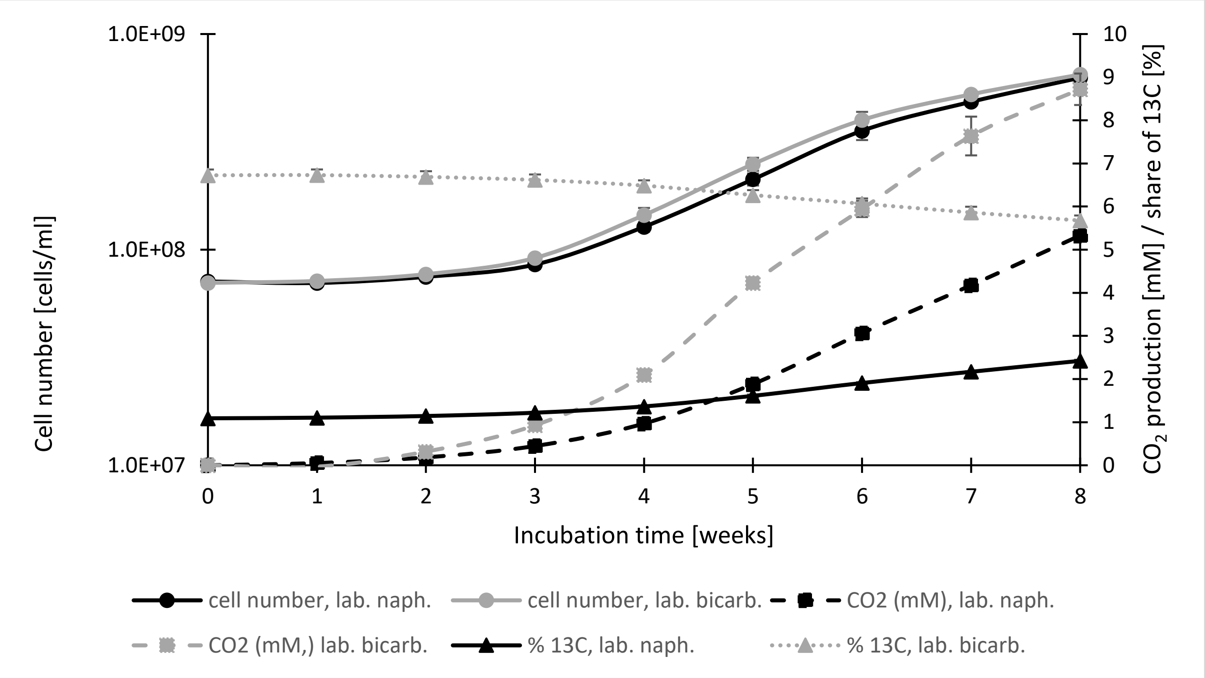

For each experiment, three separate cultures were grown under the same conditions and analyzed as triplicates, CO_2 measurements were performed as technical duplicates of each individual culture. Error bars depict standard deviations of the three data points. Black symbols and lines represent cultures with labeled naphthalene and grey cultures with labeled bicarbonate. Solid lines show the mean cell counts in cells/ml, dashed lines the mean CO_2 production in the cultures in mM and dotted lines the share of ^{13}C in the cultures in %. Error bars depict the standard deviation of the three replicate cultures and can be smaller than the symbol size so remain unseen.

The isotope ratio of the CO_2 pool in the culture medium used for CO_2-fixation equals the isotope ratio of the initial bicarbonate buffer plus the isotope ratio of the CO_2 released from oxidation of labeled or unlabeled naphthalene. Hence, the ^{13}C-content of the carbonate buffer changed over the time course of the cultivation (figure S1). Approximately 23 % of naphthalene were converted to CO_2 at the end of the experiment and the cultures with 10 % ^{13}C-naphthalene contained approximately 2.4 % ^{13}C in the total CO_2, the cultures with 10 % ^{13}C-labeled bicarbonate 5.7 % ^{13}C. The influence of isotope fractionation effects can be neglected here, as the carbon isotope fractionation during naphthalene degradation by strain N47 was reported to be -5 ‰ [1], and therefore three orders of magnitude lower than the isotope ratios we measure here.

The difference in CO_2 production between the two experiments is likely caused by the different way of measuring and calculating CO_2 production in the two setups, as one is a classical isotope labeling approach, while the experiment with labeled bicarbonate is a reverse isotope labeling approach [2].

The cell numbers in the cultures were approx. nine times higher after eight weeks than at the beginning of the experiment (see Figure S2), meaning every cell would have divided three times on average. This means that enough new biomass was build up to give conclusive information about the ^{13}C incorporation into the new biomass.

Figure S3: Overview of the TCA cycle and the WLP including the measured enzyme activities.

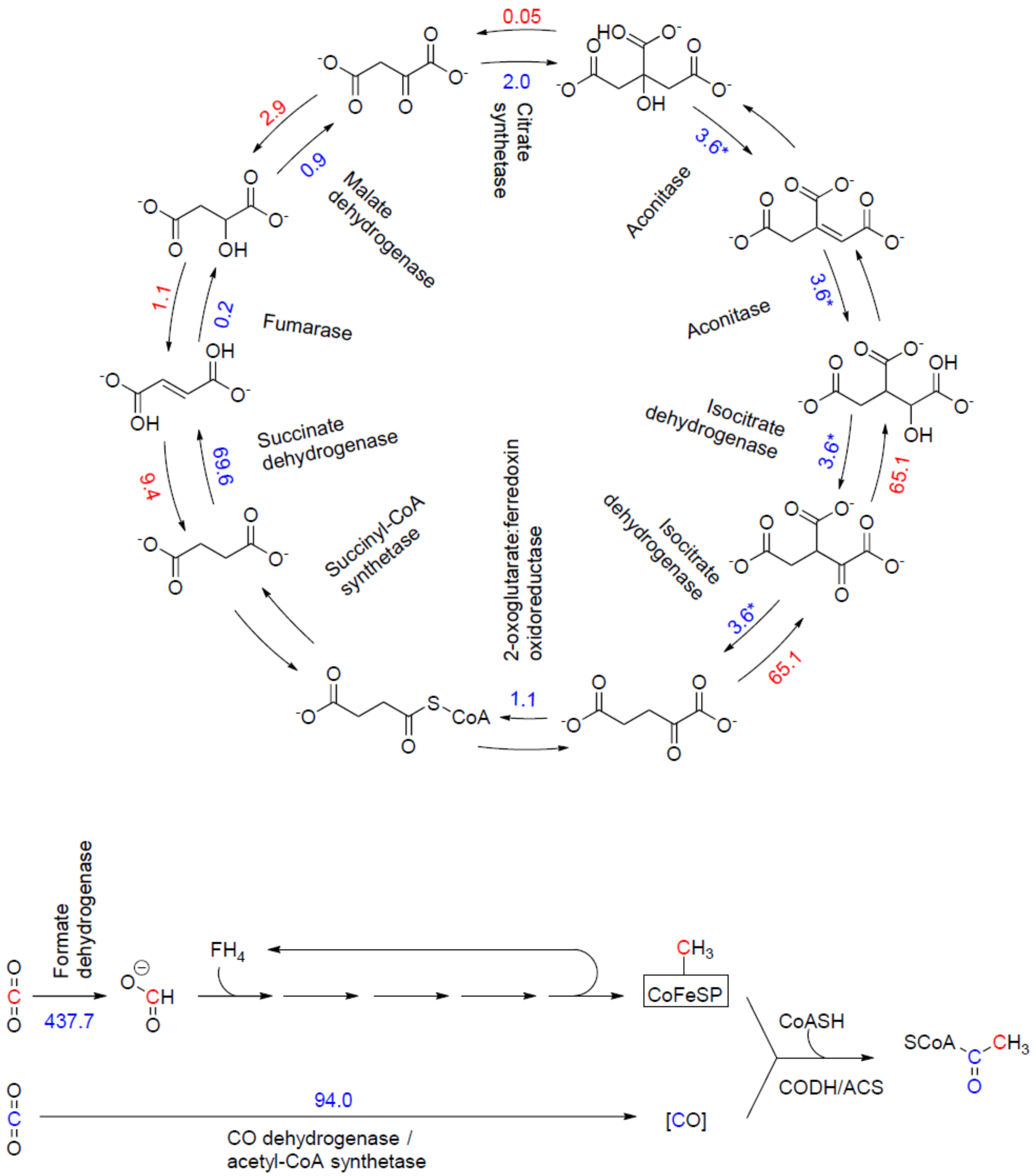

In the TCA, enzyme activities measured in the oxidative direction are labeled on the inside of the cycle in blue, activities measure in the reductive direction are labeled on the outside of the cycle in red. The activities of aconitase and isocitrate dehydrogenase could only be measured in one assay together in the oxidative direction, which is marked with an asterisk [*]. In the WLP, carbon atoms were marked in color (red in the methyl branch, blue in the carbonyl branch) to facilitate an easier overview of the fate of the carbon during the reactions. FH_4 = Tetrahydrofolate, CoFeSP = Corrinoid iron-sulfur protein, CODH/ACS = CO dehydrogenase / acetyl-CoA synthetase

Figure S4: SIM measurement of alanine.

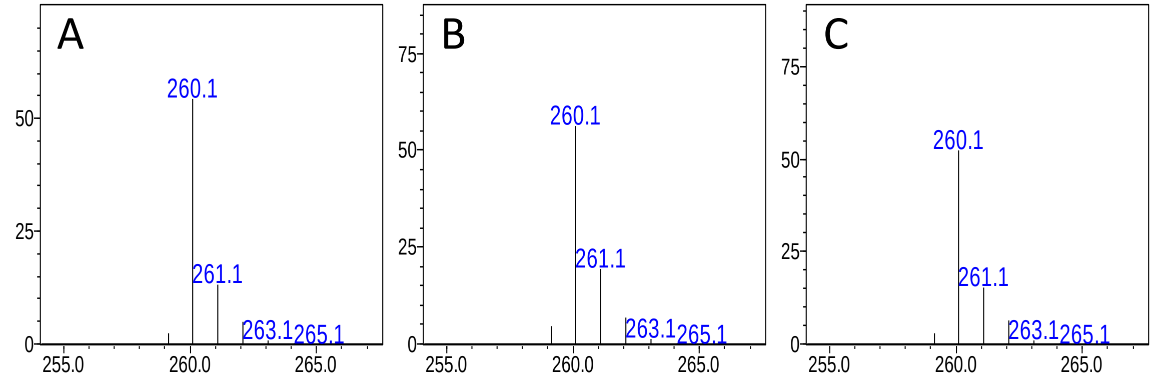

A: Experiment with no labeled precursor, B: Experiment with $^{13}CO_2$, C: Experiment with [U-^{13}C]naphthalene. Shown is the fragment [M - 57] = 260.

Figure S5: SIM measurement of aspartate.

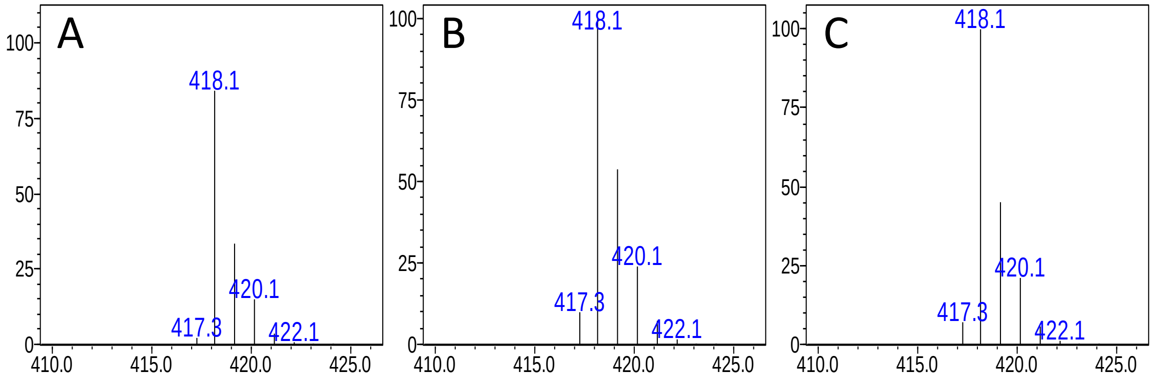

A: Experiment with no labeled precursor, B: Experiment with $^{13}CO_2$, C: Experiment with [U-^{13}C]naphthalene. Shown is the fragment [M - 57] = 418.

Figure S6: SIM measurement of glutamate.

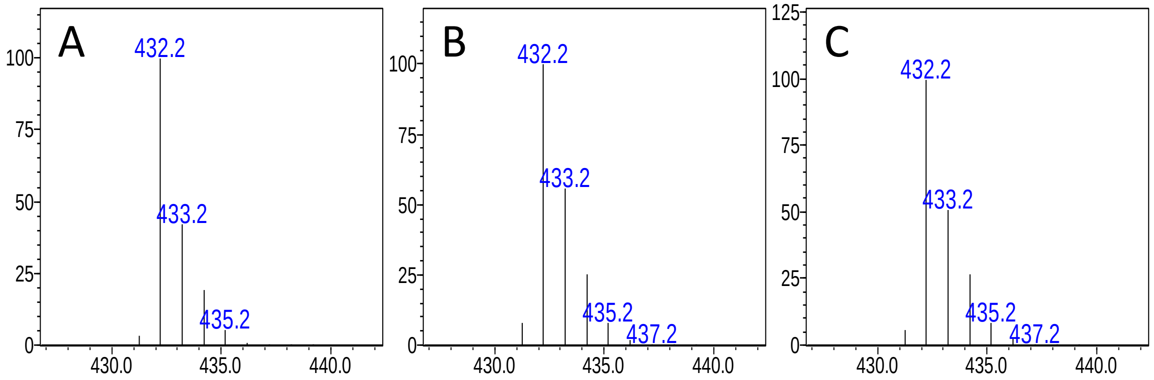

A: Experiment with no labeled precursor, B: Experiment with $^{13}CO_2$, C: Experiment with [U-^{13}C]naphthalene. Shown is the fragment [M - 57] = 432.

Figure S7: SIM measurement of FAME.

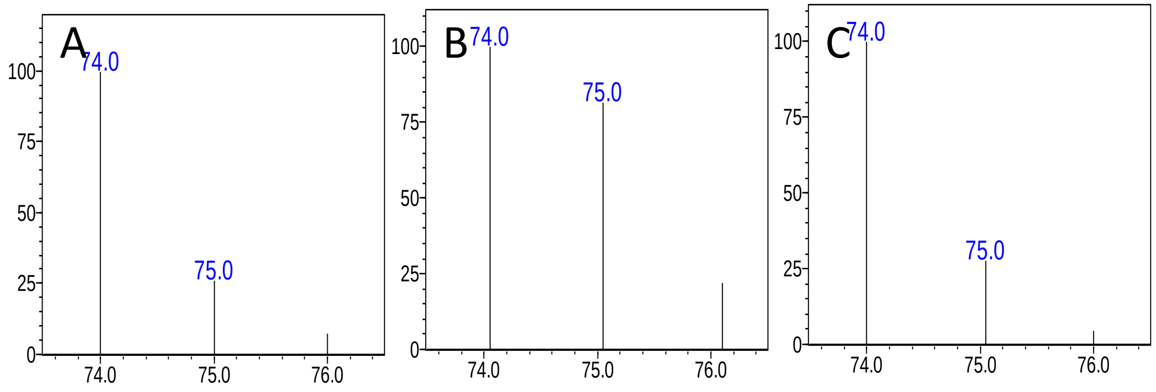

A: Experiment with no labeled precursor, B: Experiment with $^{13}CO_2$, C: Experiment with [U-^{13}C]naphthalene. Shown is the mass of the McLafferty fragment m/z = 74.

Figure S8: Typical McLafferty rearrangement of fatty acids occurring under MS-conditions.

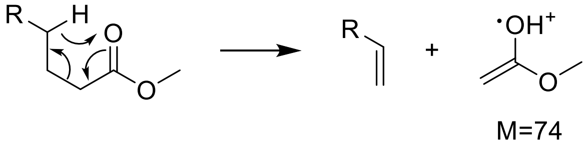

Figure S9: Fragmentation of TBDMS amino acids resulting in the M-57 fragment.

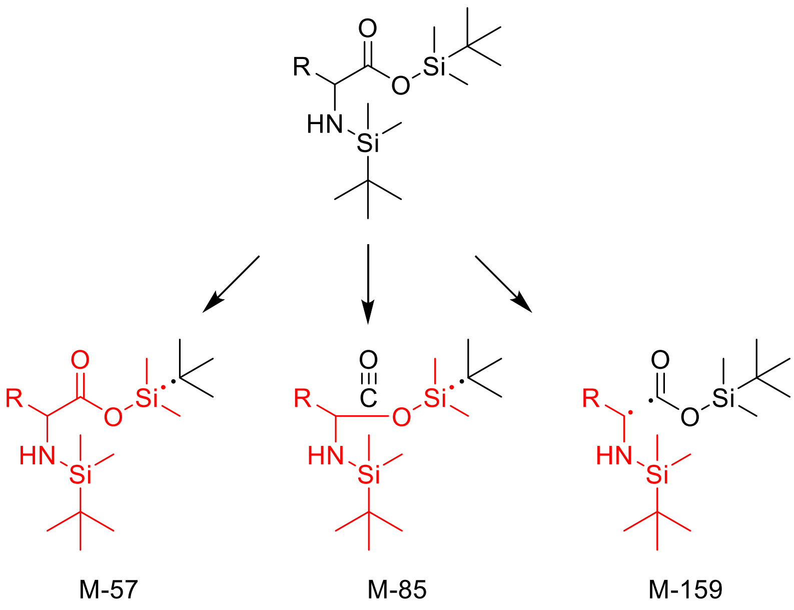

M is the mass of the molecule without any fragmentation, so e. g. “M-57” corresponds to a mass that is 57 Da lighter than the molecule was before fragmentation. Fragment M-57 results from the fragmentation of a tert-butyl group from TBDMS. The resulting fragment, which is measured by GC-MS is colored red.

Figure S10: Exemplary result of flow cytometry measurements showing the gating strategy used for counting N47 cells.

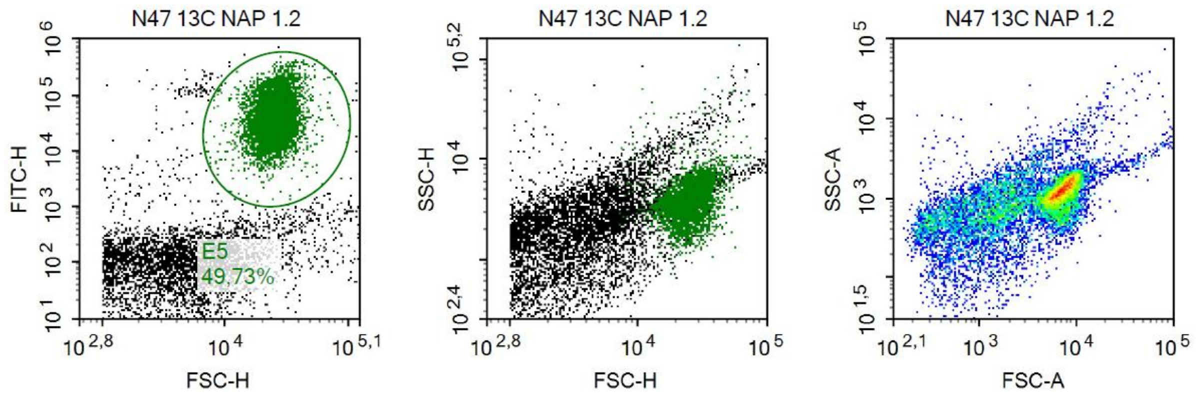

The gate was defined to distinguish N47 cells from background signals by comparing measurements of N47 cultures with measurements of sterile ultrapure water.

Supplementary table

Table S1: List of putative genes associated with the TCA cycle and the WLP in the N47 proteome and metagenome. N47 proteome was downloaded for reanalysis from GenBank, accession numbers FR695864-FR695880 [3] and metagenome was analyzed from BioProject PRJNA1066605. Most indicated genes were detected in both the proteome and metagenome. Only aconitase or aconitate hydratase was identified only in the N47 metagenome and some genes for subunits (2-oxoglutarate ferredoxin oxidoreductase subunit delta, succinate dehydrogenase flavoprotein subunit, succinate dehydrogenase iron-sulfur subunit, succinate dehydrogenase cytochrome b subunit) were not found in the metagenome. However, other subunits of those enzymes could be identified and the lack might be a result of metagenomic binning.[3]

**Name**	**Proteome:** **scaffold ID; gene start to end position in N47 genome [3]**	**GenBank locus_tag**	**Metagenome: contig ID in N47_Bin1 BioProject PRJNA1066605**
**TCA**			
CS, gltA; citrate synthase [EC:2.3.3.1]	FR695877.1: 71810-73135	N47_E41590	k141_426_31
ACO, acnA; aconitate hydratase [EC:4.2.1.3]	/	/	k141_961_7
IDH1, IDH2, icd; isocitrate dehydrogenase [EC:1.1.1.42]	FR695872.1: 579134-580360	N47_J06360	k141_632_6
korA, oorA, oforA; 2-oxoglutarate/2-oxoacid ferredoxin oxidoreductase subunit alpha [EC:1.2.7.3 1.2.7.11]	FR695864.1: 279009-280079<br>FR695872.1: 165933-166436; 301863-303014<br>FR695877.1: 899295-901001	N47_A10080<br>N47_J01860; N47_J03290<br>N47_E50970	k141_26_2<br>k141_650_4<br>k141_711_10
korB, oorB, oforB; 2-oxoglutarate/2-oxoacid ferredoxin oxidoreductase subunit beta [EC:1.2.7.3 1.2.7.11]	FR695864.1: 280153-280908<br>FR695872.1: 303017-303820<br>FR695877.1: 901015-901869	N47_A10090<br>N47_J03300<br>N47_E50980	k141_650_3<br>k141_711_9
korC, oorC; 2-oxoglutarate ferredoxin oxidoreductase subunit gamma [EC:1.2.7.3]	FR695864.1: 280908-281447<br>FR695872.1: 303823-304368	N47_A10100<br>N47_J03310	k141_650_2

korD, oorD; 2-oxoglutarate ferredoxin oxidoreductase subunit delta [EC:1.2.7.3]	FR695864.1: 278759-278989<br>FR695872.1: 165559-165825	N47_A10070<br>N47_J01850	/
sucD; succinyl-CoA synthetase alpha subunit [EC:6.2.1.5]	FR695870.1: 16728-17606	N47_B19710	k141_457_2
sucC; succinyl-CoA synthetase beta subunit [EC:6.2.1.5]	FR695870.1: 17644-18807	N47_B19720	k141_457_3
sdhA, frdA; succinate dehydrogenase flavoprotein subunit [EC:1.3.5.1]	FR695874.1: 299723-301636	N47_D30960	/
sdhB, frdB; succinate dehydrogenase iron-sulfur subunit [EC:1.3.5.1]	FR695874.1: 298962-299726<br>FR695876.1: 38311-38982	N47_D30950<br>N47_K27410	/
sdhC, frdC; succinate dehydrogenase cytochrome b subunit	FR695874.1: 301650-302291	N47_D30970	/
frdA; succinate dehydrogenase flavoprotein subunit [EC:1.3.5.1]	FR695866.1: 199754-201379	N47_H23300	k141_190_10
E4.2.1.2A, fumA, fumB; fumarate hydratase, class I [EC:4.2.1.2]	FR695866.1: 196177-197787	N47_H23280	k141_190_12
mdh; malate dehydrogenase [EC:1.1.1.37]	FR695877.1: 891276-892211	N47_E50870	k141_711_19
por, nifJ; pyruvate-ferredoxin/flavodoxin oxidoreductase [EC:1.2.7.1 1.2.7.-]	FR695874.1: 172654-176193	N47_D29660	k141_613_40
pps, ppsA; pyruvate, water dikinase [EC:2.7.9.2]	FR695870.1: 139341-141941<br>FR695872.1: 43783-46344<br>FR695872.1: 416771-419344	N47_B21030<br>N47_J00480<br>N47_J04540	k141_1080_5<br>k141_929_25<br>k141_937_8
ppdK; pyruvate, orthophosphate dikinase [EC:2.7.9.1]	FR695872.1: 589440-592178	N47_J06480	k141_632_17
pycB; pyruvate carboxylase subunit B [EC:6.4.1.1]	FR695865.1: 2992-4959<br>FR695870.1: 19248-21242	N47_I06860<br>N47_B19730	k141_457_4<br>k141_905_2

WLP			
cdhE, acsC; acetyl-CoA decarbonylase/synthase, CODH/ACS complex subunit gamma [EC:2.1.1.245]	FR695877.1: 241699-243039	N47_E43410	k141_350_9
cdhD, acsD; acetyl-CoA decarbonylase/synthase, CODH/ACS complex subunit delta [EC:2.1.1.245]	FR695877.1: 247514-249079	N47_E43440	k141_350_12
cooS, acsA; anaerobic carbon-monoxide dehydrogenase catalytic subunit [EC:1.2.7.4]	FR695874.1: 177282-179195<br>FR695877.1: 245370-247412<br>FR695877.1: 642975-644945	N47_D29680<br>N47_E43430<br>N47_E47810	k141_350_11<br>k141_613_41<br>k141_614_12
acsB; acetyl-CoA synthase [EC:2.3.1.169]	FR695877.1: 243095-245308	N47_E43420	k141_350_10
metF, MTHFR; methylenetetrahydrofolate reductase (NADH) [EC:1.5.1.54]	FR695864.1: 239599-240531<br>FR695868.1: 339597-340532; 607140-608069; 626237-627112<br>FR695874.1: 186120-186983<br>FR695877.1: 454954-455886; 738171-739094	N47_A09740<br>N47_G35960; N47_G39220; N47_G39380<br>N47_D29760<br>N47_E45610; N47_E48840	k141_388_41<br>k141_561_31<br>k141_613_49
acsE; 5-methyltetrahydrofolate corrinoid/iron sulfur protein methyltransferase [EC:2.1.1.258]	FR695877.1: 240733-241614	N47_E43400	k141_350_8

Supplementary references

1. Kanehisa, M., Furumichi, M., Sato, Y., Kawashima, M. & Ishiguro-Watanabe, M. KEGG for taxonomy-based analysis of pathways and genomes. *Nucleic Acids Res.* **51**, D587-D592 (2023). https://doi.org:10.1093/nar/gkac963
2. Dong, X. *et al.* Monitoring Microbial Mineralization Using Reverse Stable Isotope Labeling Analysis by Mid-Infrared Laser Spectroscopy. *Environ. Sci. Technol.* **51**, 11876-11883 (2017). https://doi.org:10.1021/acs.est.7b02909
3. Bergmann, F. *et al.* Genomic insights into the metabolic potential of the polycyclic aromatic hydrocarbon degrading sulfate-reducing *Deltaproteobacterium* N47. *Environ. Microbiol.* **13**, 1125-1137 (2011).
