# Supplementary material for: Chemoorganoautotrophic lifestyle of the anaerobic enrichment culture N47 growing on naphthalene
Source: Commun Biol. 2025 Jun 4;8:856. doi: 10.1038/s42003-025-08172-y (PMC12137691; doi:10.1038/s42003-025-08172-y)
Supplement: Supplementary file 2 — Description of additional supplementary materials [file 42003_2025_8172_MOESM2_ESM.pdf]

Description of Additional Supplementary Files

File name: Supplementary Data

Description: This file contains the source data for Table 1 and Table 2 of the main text.
