# Supplementary material for: Chemoorganoautotrophic lifestyle of the anaerobic enrichment culture N47 growing on naphthalene
Source: Commun Biol. 2025 Jun 4;8:856. doi: 10.1038/s42003-025-08172-y (PMC12137691; doi:10.1038/s42003-025-08172-y)
Supplement: Supplementary file 4 — Reporting summary [file 42003_2025_8172_MOESM4_ESM.pdf]

nature portfolio

Corresponding author(s): Prof. Rainer U. Meckenstock

Last updated by author(s): Mar 23, 2025

Reporting Summary

Nature Portfolio wishes to improve the reproducibility of the work that we publish. This form provides structure for consistency and transparency in reporting. For further information on Nature Portfolio policies, see our Editorial Policies and the Editorial Policy Checklist.

Statistics

For all statistical analyses, confirm that the following items are present in the figure legend, table legend, main text, or Methods section.

n/a	Confirmed	
☐	☒	The exact sample size (*n*) for each experimental group/condition, given as a discrete number and unit of measurement
☐	☒	A statement on whether measurements were taken from distinct samples or whether the same sample was measured repeatedly
☐	☒	The statistical test(s) used AND whether they are one- or two-sided<br>*Only common tests should be described solely by name; describe more complex techniques in the Methods section.*
☐	☒	A description of all covariates tested
☐	☒	A description of any assumptions or corrections, such as tests of normality and adjustment for multiple comparisons
☐	☒	A full description of the statistical parameters including central tendency (e.g. means) or other basic estimates (e.g. regression coefficient) AND variation (e.g. standard deviation) or associated estimates of uncertainty (e.g. confidence intervals)
☒	☐	For null hypothesis testing, the test statistic (e.g. *F*, *t*, *r*) with confidence intervals, effect sizes, degrees of freedom and *P* value noted<br>*Give P values as exact values whenever suitable.*
☒	☐	For Bayesian analysis, information on the choice of priors and Markov chain Monte Carlo settings
☒	☐	For hierarchical and complex designs, identification of the appropriate level for tests and full reporting of outcomes
☒	☐	Estimates of effect sizes (e.g. Cohen's *d*, Pearson's *r*), indicating how they were calculated

Our web collection on statistics for biologists contains articles on many of the points above.

Software and code

Policy information about availability of computer code

Data collection	No specific software was used for data collection except for the instrument software of the respective measuring devices, e. g. Shimadzu LabSolutions for GC-MS measurements.
Data analysis	For data analysis of the GC-MS measurements of the isotope labeled amino acids a custom excel macro was used. The calculations using this macro are the same than the calculations carried out using the freely available software "Isotopo" and are explained in the manuscript.<br>No other custom software or code was used.

For manuscripts utilizing custom algorithms or software that are central to the research but not yet described in published literature, software must be made available to editors and reviewers. We strongly encourage code deposition in a community repository (e.g. GitHub). See the Nature Portfolio guidelines for submitting code & software for further information.

Data

Policy information about availability of data

All manuscripts must include a data availability statement. This statement should provide the following information, where applicable:

- Accession codes, unique identifiers, or web links for publicly available datasets
- A description of any restrictions on data availability
- For clinical datasets or third party data, please ensure that the statement adheres to our policy

All data are available in the main text or the supplementary materials. N47 genome was used from Bergmann et al., 2011 22 and downloaded for reanalysis from

GenBank (accession numbers FR695864-FR695880, Table S1) and reanalyzed via DRAM (Distilled and Refined Annotation of Metabolism) tool 59, without installation of UniRef90 database, metabolic pathways were predicted by KEGG 44 pathway profiling of DRAM annotations. All other data are available on the KEGG online database, last search date was the 17.07.23. A new metagenome from N47 culture from March 2022 was analyzed and uploaded to the GenBank. This Whole Genome Shotgun project has been deposited at GenBank under the accession JBAMMY000000000. The version described in this paper is version JBAMMY010000000.

Research involving human participants, their data, or biological material

Policy information about studies with human participants or human data. See also policy information about sex, gender (identity/presentation), and sexual orientation and race, ethnicity and racism.

Reporting on sex and gender	*Use the terms sex (biological attribute) and gender (shaped by social and cultural circumstances) carefully in order to avoid confusing both terms. Indicate if findings apply to only one sex or gender; describe whether sex and gender were considered in study design; whether sex and/or gender was determined based on self-reporting or assigned and methods used. Provide in the source data disaggregated sex and gender data, where this information has been collected, and if consent has been obtained for sharing of individual-level data; provide overall numbers in this Reporting Summary. Please state if this information has not been collected. Report sex- and gender-based analyses where performed, justify reasons for lack of sex- and gender-based analysis.*
Reporting on race, ethnicity, or other socially relevant groupings	*Please specify the socially constructed or socially relevant categorization variable(s) used in your manuscript and explain why they were used. Please note that such variables should not be used as proxies for other socially constructed/relevant variables (for example, race or ethnicity should not be used as a proxy for socioeconomic status). Provide clear definitions of the relevant terms used, how they were provided (by the participants/respondents, the researchers, or third parties), and the method(s) used to classify people into the different categories (e.g. self-report, census or administrative data, social media data, etc.) Please provide details about how you controlled for confounding variables in your analyses.*
Population characteristics	*Describe the covariate-relevant population characteristics of the human research participants (e.g. age, genotypic information, past and current diagnosis and treatment categories). If you filled out the behavioural & social sciences study design questions and have nothing to add here, write "See above."*
Recruitment	*Describe how participants were recruited. Outline any potential self-selection bias or other biases that may be present and how these are likely to impact results.*
Ethics oversight	*Identify the organization(s) that approved the study protocol.*

Note that full information on the approval of the study protocol must also be provided in the manuscript.

Field-specific reporting

Please select the one below that is the best fit for your research. If you are not sure, read the appropriate sections before making your selection.

☒ Life sciences ☐ Behavioural & social sciences ☐ Ecological, evolutionary & environmental sciences

For a reference copy of the document with all sections, see nature.com/documents/nr-reporting-summary-flat.pdf

Life sciences study design

All studies must disclose on these points even when the disclosure is negative.

Sample size	For most experiments, experiments were carried out in triplicates. For analysis of fatty acids the triplicates had to be pooled together to have enough cell mass for the analysis.
Data exclusions	No data was excluded.
Replication	Enzyme assays were carried out in triplicates but not replicated several times due to the slow growth of the culture and the very small resulting cell yield. To compensate for this, many different controls were carried out (each in triplicates as well), in each set of controls one substrate was omitted from the assay mix. An assay was only regarded as positive if it was different from all controls. In the manuscript it is stated for each assay which control set was used for correction of the resulting activities.<br>GC-MS measurements of amino acids were carried out from three seperately grown cultures as stated in the manuscript and each was measured three times (technical replicates). For measurements of fatty acids, the three different cultures had to be pooled together to have enough cell mass for measurements, but they were also measured in technical triplicates.
Randomization	There were no "experimental groups". Triplicates were simply numbered 1 to 3 and were always treated in the same way at the same time and by the same person conducting the experiments.
Blinding	There was no blinding perfomed here. All measurements were carried out identically and by the same person for each experiment.

Reporting for specific materials, systems and methods

We require information from authors about some types of materials, experimental systems and methods used in many studies. Here, indicate whether each material, system or method listed is relevant to your study. If you are not sure if a list item applies to your research, read the appropriate section before selecting a response.

Materials & experimental systems

n/a	Involved in the study
☒	☐ Antibodies
☒	☐ Eukaryotic cell lines
☒	☐ Palaeontology and archaeology
☒	☐ Animals and other organisms
☒	☐ Clinical data
☒	☐ Dual use research of concern
☒	☐ Plants

Methods

n/a	Involved in the study
☒	☐ ChIP-seq
☐	☒ Flow cytometry
☒	☐ MRI-based neuroimaging

Plants

Seed stocks	*Report on the source of all seed stocks or other plant material used. If applicable, state the seed stock centre and catalogue number. If plant specimens were collected from the field, describe the collection location, date and sampling procedures.*
Novel plant genotypes	*Describe the methods by which all novel plant genotypes were produced. This includes those generated by transgenic approaches, gene editing, chemical/radiation-based mutagenesis and hybridization. For transgenic lines, describe the transformation method, the number of independent lines analyzed and the generation upon which experiments were performed. For gene-edited lines, describe the editor used, the endogenous sequence targeted for editing, the targeting guide RNA sequence (if applicable) and how the editor was applied.*
Authentication	*Describe any authentication procedures for each seed stock used or novel genotype generated. Describe any experiments used to assess the effect of a mutation and, where applicable, how potential secondary effects (e.g. second site T-DNA insertions, mosiacism, off-target gene editing) were examined.*

Flow Cytometry

Plots

Confirm that:

☒ The axis labels state the marker and fluorochrome used (e.g. CD4-FITC).

☒ The axis scales are clearly visible. Include numbers along axes only for bottom left plot of group (a 'group' is an analysis of identical markers).

☐ All plots are contour plots with outliers or pseudocolor plots.

☐ A numerical value for number of cells or percentage (with statistics) is provided.

Methodology

Sample preparation	Cells were sampled anoxically with a sterile syringe from the anoxic culture bottles. The sample was diluted 1:1000 with sterile ultrapure water and stained with 5µM Syto9 in sterile reaction tubes for 30min in the dark before measurement.
Instrument	NovoCyte 2000R, Agilent
Software	Instrument software: NovoExpress Version 1.6.2
Cell population abundance	The culture was regarded as a pure culture for this study. It contains approx. 3% other (but closely related) cells, but these cannot be distinguished from N47 cells during flow cytometry.
Gating strategy	Gating was used to distinguish N47 cells from the background signal. The gate was defined experimentally by comparing counts of visibly grown N4 cultures to counts of sterile ultrapure water and setting the gate to count only the cells in N47 in areas of the plot that are blank in the ultrapure water samples. A figure is provided in the supplementary materials.

☒ Tick this box to confirm that a figure exemplifying the gating strategy is provided in the Supplementary Information.
